# The Role of Endoplasmic Reticulum Stress-Glycogen Synthase Kinase-3 Signaling in Atherogenesis

**DOI:** 10.3390/ijms19061607

**Published:** 2018-05-30

**Authors:** Aric Huang, Sarvatit Patel, Cameron S. McAlpine, Geoff H. Werstuck

**Affiliations:** 1 Thrombosis and Atherosclerosis Research Institute, McMaster University, Hamilton, ON L9L 2X2, Canada; aric_huang_92@hotmail.com (A.H.); sarvatit.patel@taari.ca (S.P.); mcalpine.cameron@gmail.com (C.S.M.); 2 Department of Chemistry and Chemical Biology, McMaster University, Hamilton, ON L8S 4L8, Canada; 3 Department of Medicine, McMaster University, 1280 Main St W, Hamilton, ON L8S 4L8, Canada

**Keywords:** atherosclerosis, risk factors, molecular mechanisms, endoplasmic reticulum (ER)-stress, glycogen synthase kinase (GSK)-3

## Abstract

Cardiovascular disease (CVD) is the number one cause of global mortality and atherosclerosis is the underlying cause of most CVD. However, the molecular mechanisms by which cardiovascular risk factors promote the development of atherosclerosis are not well understood. The development of new efficient therapies to directly block or slow disease progression will require a better understanding of these mechanisms. Accumulating evidence supports a role for endoplasmic reticulum (ER) stress in all stages of the developing atherosclerotic lesion however, it was not clear how ER stress may contribute to disease progression. Recent findings have shown that ER stress signaling through glycogen synthase kinase (GSK)-3α may significantly contribute to macrophage lipid accumulation, inflammatory cytokine production and M1macrophage polarization. In this review we summarize our knowledge of the potential role of ER stress-GSK3 signaling in the development and progression of atherosclerosis as well as the possible therapeutic implications of this pathway.

## 1. Introduction

Cardiovascular diseases (CVDs) are a group of disorders involving the heart and blood vessels. They are the leading cause of mortality worldwide accounting for approximately 32% of all deaths (17.9 million deaths in 2015) [1]. This number is expected to increase to over 23.6 million deaths per year by 2030. Therefore, CVDs represent a major burden on health care systems around the world. Risk factors for CVDs include hypertension (resting blood pressure >140/90 mmHg), dyslipidemia (total cholesterol >240 mg/dL, low-density lipoprotein (LDL) >160 mg/dL, and high density lipoprotein (HDL) <40 mg/dL), physical inactivity, obesity (body mass index >30 kg/m^2^), diabetes mellitus (elevated blood glucose, fasting glucose level ≥126 mg/dL, or 2 h plasma glucose during oral glucose tolerance test ≥200 mg/dL with a loading dose of 75 g, or random blood glucose level ≥200 mg/dL, or glycated hemoglobin A1C ≥6.5%), and tobacco use [2]. Non-modifiable risk factors include advancing age, ethnicity and family history.

Over the last few decades, significant advances have been made in the diagnosis, prevention and treatment of CVDs. Current therapies target the risk factors (hypertension, diabetes, dyslipidemia) as opposed to the disease itself because our knowledge of the underlying molecular mechanisms and pathways by which these cardiovascular risk factors promote atherosclerosis are still poorly understood. A better understanding of the molecular mechanisms that link risk factors to accelerated atherogenesis will provide novel targets for the development of new therapeutic strategies that act to directly slow, block, or even reverse disease progression.

## 2. Atherosclerosis

Healthy arteries provide a dynamic and responsive conduit for oxygen- and nutrient-rich blood to all tissues of the body, and their maintenance and repair is essential to sustain an efficient circulatory system. Atherosclerosis is an inflammatory disease characterized by the accumulation of fatty lesions or plaques in the walls of medium and large arteries that can ultimately impede blood circulation. It is the major underlying cause of cardiovascular disease [3].

**Figure 1:**
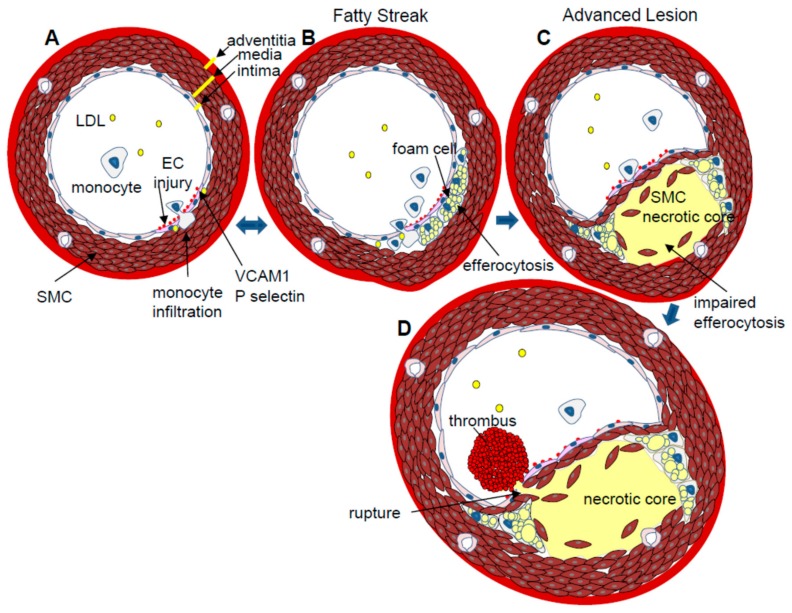
Atherogenesis. (**A**) EC Injury: Atherosclerosis is initiated at sites of EC injury. Damaged ECs express and present adhesive molecules, VCAM-1 and P-selectin, that facilitate the accumulation of circulating monocytes and T cells at the site of vessel wall damage. Monocytes move into the sub-endothelial intima where they differentiate into macrophages; (**B**) Fatty streak: macrophages endocytose oxidized-LDL particles and apoptotic cell bodies (efferocytosis), becoming lipid-engorged foam cells. Activated lesional macrophages also secrete pro-inflammatory cytokines, including IFNγ, IL1β and TNFα; (**C**) Advanced plaque: cytokines and chemokines induce vascular smooth muscle cells (SMCs) to migrate from the media to the intima where they secrete collagen fibres that form a fibrous cap over the growing plaque. If macrophage foam cell apoptosis exceeds the rate of efferocytosis, an acellular, cholesterol-rich necrotic core will form and destabilize the lesion. Matrix metaloproteases that digest the protective fibrous cap can further destabilize the atherosclerotic plaque; (**D**) Plaque rupture: if an atherosclerotic plaque ruptures, the circulating blood comes into contact with the necrotic core, resulting in the formation of a thrombus that can occlude the artery and cause a myocardial infarction or stroke.

The artery wall is made up of three distinct layers; the tunica intima, tunica media and adventitia. The intima consists of a single layer of endothelial cells that acts as the interface between the circulating blood and the rest of the vessel wall. Endothelial cells are very responsive to blood flow characteristics. Regions of the endothelium exposed to high shear stress and laminar blood flow are relatively protected from atherosclerosis. In regions of the vascular wall that are exposed to turbulent, non-laminar blood flow, including bifurcations, branches and inner curvatures, endothelial cells chronically express increased levels of inflammatory factors [4]. These regions of non-laminar blood flow appear to be more prone to insult or injury that may result from exposure to agents that further challenge the endothelium, including elevated VLDL/LDL levels, high glucose concentrations, hypertension, and blood-borne toxins from cigarette smoke. In response to injury, affected endothelial cells express surface proteins, including vascular cell adhesion molecule (VCAM)-1 and P-selectin, which promote the accumulation of monocytes at the site of damage [5,6]. Monocytes, T lymphocytes and other leukocytes migrate across the endothelium into the sub-endothelial intimal layer (Figure 1). Once in the sub-endothelial space, monocytes differentiate into macrophages, which internalize apolipoprotein B-containing lipoproteins (VLDL and LDL) and especially oxidized-LDL particles that have entered the subendothelial space. The lipid engorged macrophages, known as foam cells, make up the fatty streak in the artery wall, which is the earliest type of discernible atherosclerotic lesion [7]. Macrophage foam cells also secrete pro-inflammatory cytokines, including interferon (IFN)-γ, interleukin (IL)-1β, and tumor necrosis factor (TNF)-α, as well as chemokines which enhance the recruitment of additional monocytes and T cells into the growing lesion [8]. Recently it has become evident that many lesional macrophages, especially in more advanced lesions, are derived from macrophage proliferation within the plaque, as opposed to differentiation of recruited monocytes [9]. Other studies have shown that many lesional macrophages are derived from a smooth muscle cell lineage [10]. The potential phenotypic differences, and the corresponding impact on atherogenesis, of these differentially derived macrophage/foam cells are not yet known.

Macrophages make up the majority of the cellular composition of a typical plaque and they play a central role in all stages of atherosclerotic plaque development and progression. Conditions within the lesional microenvironment determine macrophage phenotypic polarization during plaque development. Pro-inflammatory M1 (classical) and anti-inflammatory M2 (alternative) macrophages represent the extreme phenotypes of a continuum of macrophage subtypes that are actually found in an atherosclerotic lesion [8]. M1 macrophages appear to play an important role early in lesion development by initiating the response to injury through the production of inflammatory cytokines [11,12]. M2 macrophages are more efficient at endocytosing apoptotic bodies (efferocytosis) and also secrete anti-inflammatory cytokines (IL4, IL10), which are likely important in the resolution of the inflammatory response [12]. It is generally accepted that the accumulation of intimal macrophage/foam cells is a normal response to injury and part of the normal maintenance of the artery wall. Under ideal conditions, macrophage/foam cell efferocytosis limits the growth of the lesion and maintains its dynamic, cellular nature. Macrophage/foam cells can actively transfer lipids onto high density lipoprotein particles (HDL) through the process of reverse cholesterol transport [13]. HDL particles carry cholesterol away from the lesion and back to the liver. Experimental evidence suggests that unburdened macrophages can move out of the artery wall, thereby completing the artery repair process [14].

In some cases, for reasons that are not fully understood, unresolved inflammation continues to stimulate the growth of the lesion and impaired efferocytosis disrupts the ability of macrophages to phagocytose apoptotic cells. As the lesion grows, foam cells continue to take up modified-LDL particles. The accumulation of free cholesterol in the foam cells can ultimately initiate apoptosis [15]. Apoptotic macrophage-derived cell bodies that are not efficiently cleared undergo a process called secondary necrosis, resulting in the formation of an acellular, lipid-rich, necrotic core within the lesion. Necrosis is a key feature of advanced, unstable plaques that are prone to rupture.

In advanced atherosclerosis, accumulating macrophage foam cells and lesional leukocytes continue to amplify the inflammatory response by secreting cytokines and growth factors. These cytokines induce the migration of vascular smooth muscle cells (VSMCs) from the tunica media into the intima [16]. Advanced lesions are characterized by a fibrous cap, containing VSMC and macrophage-synthesized collagen, which covers and stabilizes the lipid core. Macrophage/foam cells also secrete collagenases, including matrix metalloproteinase (MMP)-1, -2, and -9, which disrupt the biomechanical stability of the fibrous cap [17]. The thinning of the fibrous cap contributes to the destabilization of the lesion, making it more susceptible to rupture. If the lesion ruptures, coagulation factors in the blood come into contact with pro-coagulant proteins, including tissue factor, in the necrotic core. This promotes platelet aggregation and the thrombosis superimposed upon the atherosclerotic plaque (atherothrombosis) [18]. The thrombus may occlude the artery, resulting in cardiovascular complications including myocardial ischemia or infarction. Plaque rupture is the underlying cause of most myocardial infarctions [18]. To facilitate the comparative analysis of disease progression, the stages of atherosclerosis have been graded according to observed morphology as described by Stary and colleagues [7].

## 3. Molecular Mechanisms that Promote Atherosclerosis

A fine balance of stimuli, involving inflammation, monocyte recruitment, macrophage polarization, efferocytosis and resolution of inflammation, are required to repair and maintain the artery wall. An imbalance in one or more of these mechanisms can disrupt the process and lead to the development of an unstable advanced atherosclerotic plaque. The presence of cardiovascular risk factors likely tip this balance toward the disease state; however, the mechanisms by which they do this are not well understood. Knowledge of the molecular mechanisms that link risk factors to cardiovascular events is essential to the development of new strategies to slow or reverse disease progression.

## 4. The Endoplasmic Reticulum (ER) and ER Stress

**Figure 2:**
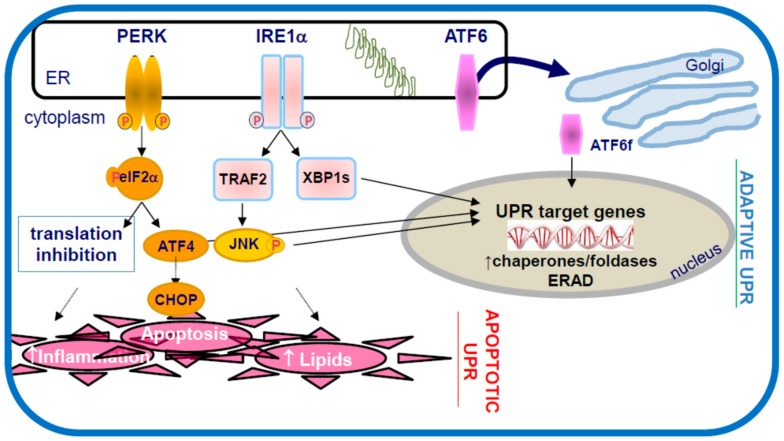
The unfolded protein response to ER stress. The accumulation of misfolded proteins in the endoplasmic reticulum, defined as ER stress, activates three ER transmembrane signaling factors PERK, IRE1 and ATF6, which initiate the unfolded protein response (UPR). Initially the adaptive UPR acts to reestablish ER homeostasis by decreasing protein flux into the ER (translation block), increasing the folding capacity of the ER (increased chaperone expression) and enhancing the ER associated protein degradation (ERAD) pathways. Chronic ER stress results in the activation of C/EBP homologous protein (CHOP) and the pro-apoptotic UPR, which can include pro-inflammatory responses and lipid accumulation—hallmark features of atherosclerosis.

The endoplasmic reticulum (ER) is a eukaryotic organelle responsible for protein modification, folding, and trafficking. A disturbance in ER function results in an accumulation of misfolded proteins, a condition known as ER stress (Figure 2). The unfolded protein response (UPR) is a cellular self-defence mechanism to help alleviate the problem and restore ER homeostasis [19,20,21]. The UPR consists of three main signaling pathways, each initiated and regulated by an ER transmembrane sensor/signaling protein; protein kinase RNA-like ER kinase (PERK), inositol-requiring enzyme-1 (IRE1), and activating transcription factor (ATF)-6. Together, these signaling pathways contribute to the adaptive response of the UPR, where the cell inhibits general protein synthesis, increases ER folding capacity by enhancing ER chaperone expression, and promotes the degradation of irreversibly misfolded proteins. If the ER stress persists, the UPR upregulates pro-apoptotic factors including the C/EBP homologous protein (CHOP) to mediate apoptosis to eliminate the cell [22]. Crosstalk between the three UPR pathways is believed to facilitate a coordinated response to conditions of ER stress. While the proximal responses to ER stress have been examined in some detail, the more distal implications of chronic ER stress are less well understood. Interestingly, ER stress and/or UPR activation have been implicated in several diseases and disorders including neurodegenerative diseases (Alzheimer’s, Amyotrophic lateral sclerosis, Huntington’s, Parkinson’s), diabetes (insulin resistance, beta cell dysfunction), non-alcoholic hepatic steatosis, cancer, kidney diseases, and others [23].

## 5. ER Stress and Atherosclerosis

It is now well established that ER stress plays a direct role in the development of atherosclerosis and UPR activation can be detected at all stages of atherosclerotic progression [20,24,25,26,27]. Risk factors for CVD including, hyperhomocysteinemia [28], obesity [29], dyslipidemia [30,31], hypertension [32], and cigarette smoke [33], have been linked to increased ER stress in the arterial wall of mouse model systems and indications of UPR activation have been observed in diseased arteries from human patients [34]. Elevated concentrations of unesterified cholesterol, palmitic acid, glucose and toxins associated with cigarette smoke have been shown to disrupt ER homeostasis and induce ER stress/UPR activation in cultured ECs, VSMC and/or macrophages.

Results from experimental interventions targeting ER stress or the UPR also support a role for this pathway in atherogenesis. Apolipoprotein E knockout (ApoE^−/−^) and LDL receptor knockout (LDLR^−/−^) mice deficient in the pro-apoptotic UPR gene, CHOP, have significantly smaller necrotic cores as well as reductions in atherosclerotic lesion area at the aortic sinus [35]. Systemic administration of chemical chaperones, 4-phenylbutyrate (4PBA) or tauroursodeoxycholic acid (TUDCA), have been shown to reduce ER stress levels and also reduced intima-to-media ratio in a wire injury model in wild type mice [36]. In both LDLR^−/−^ and ApoE^−/−^ mouse models, systemic treatment with 4PBA or TUDCA have been shown to significantly attenuate atherosclerotic progression [37,38,39]. Together, these findings suggest that ER stress plays a significant role in promoting pro-atherosclerotic processes.

The mechanisms by which ER stress promotes atherosclerosis are still being delineated. Experiments performed in vitro have demonstrated that ER stress-inducing agents can promote lipid accumulation in macrophages, hepatocytes, and other cell types by activating/dysregulating the sterol regulatory element binding proteins (SREBP1 and 2) [28,40,41]. SREBP1/2 are transcription factors that regulate the expression of genes encoding the LDLR as well as genes encoding proteins involved in cholesterol and fatty acid biosynthesis. ER stress can also activate nuclear factor (NF)-κB, a transcription factor responsible for the upregulation of inflammatory cytokines including TNFα and IL-6 [42,43]. Finally, it is well established that ER stress can promote apoptosis in various cell types, including endothelial and macrophage/foam cells, by activating the JNK/p38MAPK pathways [44,45,46]. The dysregulation of lipid metabolism and lipid accumulation, increased inflammation, and apoptosis are hallmark features of atherosclerosis. However, the precise molecular mechanisms by which chronic ER stress and/or the activation of the UPR can promote these pro-atherosclerotic responses are not understood.

## 6. Glycogen Synthase Kinase (GSK)-3

Recent findings from our laboratory suggest that ER stress may signal through glycogen synthase kinase (GSK)-3 to promote atherosclerosis [47,48,49]. GSK3α (51 kDa) and GSK3β (47 kDa), are serine/threonine kinases that are expressed ubiquitously in mammals [50]. GSK3α/β are involved in several metabolic pathways, and they have been linked to a number of diseases including Alzheimer’s disease [51], diabetes [52], and bipolar mood disorder [53]. The kinase domains of GSK3α/β are 98% homologous and there is broad overlap in substrate specificity [54]. However, GSK3α and β are not functionally equivalent, and it is now clear that the α and β forms of GSK3 have distinct functions. The most striking difference is illustrated by the fact that mice lacking GSK3α are viable, fertile and have no overt phenotype [55,56]. In contrast, GSK3β-deficient mice die of liver failure and heart defects during mid-gestation (E13.5-16.5) [57,58].

GSK3α and β are different from many other kinases because they exhibit a significant level of constitutive activity in resting cells. This can be further enhanced, or inhibited, by several upstream signaling mechanisms involving mitogens and growth factors, as well as conditions of cellular stress, including heat shock, oxidative stress, and ER stress [59,60,61]. Regulation of GSK3α/β activity is thought to predominantly occur by phosphorylation and dephosphorylation of specific residues, but is also controlled by interactions with a number of different scaffold proteins, as well as regulation of its intracellular localization [62]. The hierarchical relationships between these regulatory pathways are still being worked out.

## 7. GSK3 and Atherosclerosis

**Figure 3:**
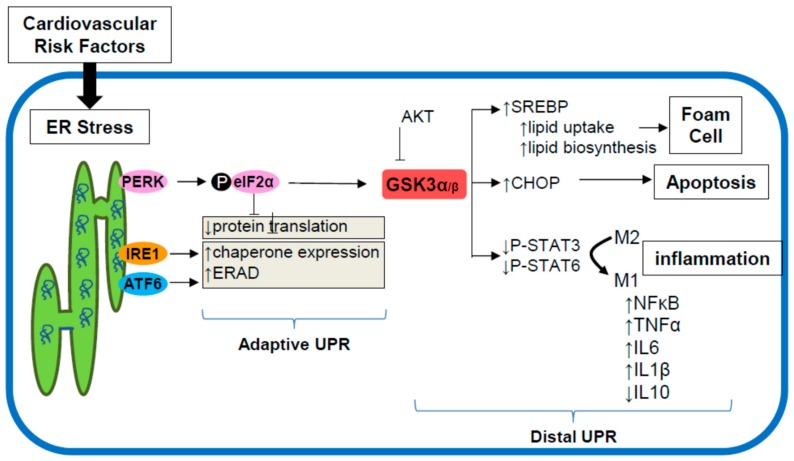
Potential mechanism by which macrophage ER stress GSK3α signaling promotes atherosclerosis. Multiple cardiovascular risk factors promote ER stress which leads to the activation of the adaptive UPR. PERK signaling can increase GSK3α/β activity. Evidence from our lab suggests that signaling through GSK3α can promote macrophage foam cell formation, activate inflammatory cytokine production and enhance CHOP expression leading to apoptosis. In support of this model, we have shown that GSK3α-deficiency in macrophages is associated with attenuated atherosclerosis.

Several lines of evidence have implicated GSK3α/β in the development of atherosclerosis (Figure 3). High fat diet (HFD) fed LDLR^−/−^ mice lacking GSK3α develop significantly smaller atherosclerotic lesions compared to LDLR^−/−^GSK3α^+/+^ controls [49]. GSK3α-deficiency also protects against HFD-induced hepatic steatosis. When fed a standard chow diet, these mice are phenotypically indistinguishable from LDLR^−/−^ control mice. Myeloid-specific deficiency of GSK3α, but not GSK3β, significantly attenuates atherogenesis in HFD-fed LDLR^−/−^ mice [63]. In vitro and in vivo analysis suggests that GSK3α plays a role in M1 macrophage polarization. This is one of the first, and certainly the most impactful, phenotype that has been specifically associated with GSK3α. Hepatic-deficiency of either GSK3α or GSK3β does not affect atherosclerotic development or liver morphology. Interestingly, a unique role for GSK3α and GSK3β has also been suggested in the polarization of CD4^+^ T cells [64]. In cultured lymphocytes, inhibition of GSK3α/β by lithium or CT99021 prevents the polarization of CD4^+^ T cells into IFN-γ producing T helper (Th)-1 cells [64]. This effect is specific to the polarization of Th1 cells as GSK3α/β inhibition does not alter Th17 or Th2 cell polarization. Moreover, when probing GSK3α and GSK3β homolog specific functions, it was noted that deletion of GSK3α, but not GSK3β, attenuated Th1 cell polarization. These observations are consistent with our observations of GSK3α, both directly and indirectly mediating macrophage M1 polarization. Furthermore, bone marrow deficiency of AKT, an upstream regulator of GSK3α/β, has been found to modulate atherosclerosis development and macrophage polarization in mice [65]. Together these findings strongly support a specific and direct role for GSK3α in the development of atherosclerosis. A number of other studies suggest that GSK3α/β may also play a pivotal role in cardiac myocyte growth and metabolism [66,67,68,69,70].

The downstream substrates through which GSK3α/β potentially regulate pro-atherogenic pathways in macrophages are not well defined. Recent evidence suggests a role for GSK3α/β signaling in the regulation of STAT phosphorylation [63,71,72,73]. GSK3α-deficient bone marrow- derived macrophages exhibit increased phosphorylation/activation of STAT3 (p-Tyr705) and STAT6 (p-Try641). Activated STAT3 inhibits STAT1, which promotes the transcription of the M1 gene program [74,75]. Activated STAT6 promotes the upregulation of the M2 gene program [74,75]. Together these results suggest that ER stress, or other signaling through GSK3α, may alter the balance of M1/M2 macrophages in the growing atherosclerotic lesion—favoring the pro-inflammatory, pro-atherosclerotic M1 polarization. The mechanism by which GSK3α regulates STAT3/6 is unclear, though it is likely indirect, as STATs are mainly regulated by tyrosine-phosphorylation. Further investigations are required to delineate direct upstream and downstream factors that link GSK3α/β to the JAK/STAT signaling pathways, as well as their role in the regulation of macrophage phenotype, particularly in the context of atherosclerosis.

## 8. ER Stress Signaling through GSK3α/β

ER stress-inducing agents can promote GSK3α/β activity, however genetic deficiency or pharmacological inhibition of GSK3α/β does not appear to alter the proximal adaptive UPR to ER stress [48,59,76]. These observations suggest that GSK3α/β lies downstream of the proximal UPR. However, GSK3α/β does contribute to ER stress-induced PERK signaling to upregulate the transcription factors ATF4 and CHOP [76]. Furthermore, pharmacological inhibition of GSK3α/β attenuates the ER stress-induced uptake of free and modified cholesterol, as well as the expression of genes involved in regulating lipid and cholesterol metabolism, such as fatty acid synthase (FAS), SREBP-1c, SREBP-2,3-hydroxy-3-methylglutaryl-coenzyme A (HMG-CoA) reductase, and the LDLR [76]. GSK3α/β can regulate the expression of several pro-inflammatory cytokines including IL-6, IL-1β, and tumor necrosis factor TNF-α through activation of nuclear factor NF-κB [57,71]. Moreover, inhibiting GSK3α/β reduces the expression of pro-inflammatory cytokines and augments anti-inflammatory cytokine production such as IL-10. These findings suggest a role of ER stress-GSK3α/β signaling in cell survival, and the formation of macrophage foam cells, and the production of inflammatory cytokines.

## 9. Targeting the ER Stress-GSK3α/β Pathway

Pre-clinical studies do support the concept of targeting various aspects of the ER stress-GSK3α/β pathways as a strategy to block or delay atherogenesis. Efforts to develop methods to manipulate the UPR have already begun, especially with respect to other diseases and disorders in which ER stress is thought to play a role. At least three general approaches have been used to address this problem. One approach is to reduce ER stress levels directly, through the addition of exogenous chemical chaperones, like 4PBA or TUDCA. Chemical chaperones have been shown to protect against ER stress associated neurodegenerative apoptosis [77], pancreatic β cell death [78], leptin resistance [79] and insulin resistance [80]. While treatment with chemical chaperones to reduced ER stress levels has proven to be efficacious at impeding atherosclerotic development in mouse models, the effective concentrations of these compounds that are required is too high to be clinically reasonable. A second approach to reduce ER stress levels is to augment specific protective aspects of the UPR to more efficiently deal with unfolded proteins. This has been accomplished by over expression of ER resident chaperones including GRP78 and calreticulin [28,81] or by treatment with a small molecule inducer of endogenous GRP78 expression, BIX [82]. This strategy is currently limited by the lack of small molecules capable of activating the adaptive UPR in vivo. A third approach is to target specific downstream factors that act to signal some of the detrimental downstream effects of ER stress, including GSK3α. Lithium and valproate are relatively non-specific GSK3α/β inhibitors. Both of these compounds have been shown to attenuate atherosclerosis in murine models [27,38,48,83]. However, these treatments have many off target side effects. While GSK3α appears to be an ideal target, there are no currently known inhibitors that can distinguish between GSK3α and β.

## 10. Conclusions

CVD continues to be a major cause of global mortality and morbidity and more effective treatments and preventative strategies are required to combat this disease. A more complete understanding of the molecular mechanisms and pathways that link CVD risk factors to the development and progression of atherosclerosis will illuminate new potential targets for drug interventions. Recent findings from our lab, and others, have shown that risk factors including dyslipidemia, obesity, diabetes, hypertension and cigarette smoke can promote ER stress in the walls of major arteries. ER stress signaling through GSK3 can activate pro-atherogenic pathways involving lipid accumulation, inflammation and apoptosis. In the near future, it will be important to continue to test and validate the contribution of ER stress-GSK3α/β signaling to accelerated atherosclerosis, in both in vivo and in vitro systems. It will be especially important to explore the possible effects or new interventions on atherosclerotic regression. An increased understanding of this pathway may lead to the establishment of novel intervention strategies for future anti-atherosclerotic drug development.

## Abbreviations

ApoE	Apolipoprotein E
ATF6	Activating transcription factor 6
CHOP	C/EBP homologous protein
CVD	Cardiovascular disease
EC	Endothelial cell
ER	Endoplasmic reticulum
ERAD	ER stress associated protein degradation
GSK3α/β	Glycogen synthase kinase 3α/β
HDL	High-density lipoprotein
HFD	High fat diet
IFN γ	Interferon γ
IL	Interleukin
IRE1	Inositol-requiring enzyme-1
JAK	Janus kinase
JNK	c-Jun N-terminal kinase
LDL	Low-density lipoprotein
LDLR	Low-density lipoprotein receptor
MAPK	Mitogen-activated protein kinase
MMP	Matrix metalloprotease
NF κB	Nuclear factor κB
4PBA	4-phenylbutyrate
PERK	Protein kinase RNA-like ER kinase
SREBP	Sterol element binding protein
STAT	Signal transducer and activator of transcription protein
Th	T helper cell
TNFα	Tumor necrosis factor α
TUDCA	Tauroursodeoxycholic acid
UPR	Unfolded protein response
VCAM1	Viral cell adhesion protein 1
VLDL	Very low-density lipoprotein
VSMC	Vascular smooth muscle cell
